# Fear Detection in Multimodal Affective Computing: Physiological Signals versus Catecholamine Concentration

**DOI:** 10.3390/s22114023

**Published:** 2022-05-26

**Authors:** Laura Gutiérrez-Martín, Elena Romero-Perales, Clara Sainz de Baranda Andújar, Manuel F. Canabal-Benito, Gema Esther Rodríguez-Ramos, Rafael Toro-Flores, Susana López-Ongil, Celia López-Ongil

**Affiliations:** 1 UC3M4Safety Team, Universidad Carlos III de Madrid, c/Butarque, 15, 28911 Madrid, Spain; lagutier@ing.uc3m.es (L.G.-M.); eleromer@ing.uc3m.es (E.R.-P.); cbaranda@hum.uc3m.es (C.S.d.B.A.); mcanabal@ing.uc3m.es (M.F.C.-B.); gerodrig@pa.uc3m.es (G.E.R.-R.); 2 Departamento de Tecnología Electrónica, c/Butarque, 15, 28911 Madrid, Spain; 3 Departamento de Comunicación, c/Madrid, 126, 28903 Madrid, Spain; 4 Fundación para la Investigación Biomédica del Hospital Universitario Príncipe de Asturias, Ctra, Alcalá-Meco s/n, 28805 Madrid, Spain; rafael.toro@uah.es (R.T.-F.); slorgil@salud.madrid.org (S.L.-O.)

**Keywords:** multimodal affective computing, catecholamines, emotion classification, wearable devices

## Abstract

Affective computing through physiological signals monitoring is currently a hot topic in the scientific literature, but also in the industry. Many wearable devices are being developed for health or wellness tracking during daily life or sports activity. Likewise, other applications are being proposed for the early detection of risk situations involving sexual or violent aggressions, with the identification of panic or fear emotions. The use of other sources of information, such as video or audio signals will make multimodal affective computing a more powerful tool for emotion classification, improving the detection capability. There are other biological elements that have not been explored yet and that could provide additional information to better disentangle negative emotions, such as fear or panic. Catecholamines are hormones produced by the adrenal glands, two small glands located above the kidneys. These hormones are released in the body in response to physical or emotional stress. The main catecholamines, namely adrenaline, noradrenaline and dopamine have been analysed, as well as four physiological variables: skin temperature, electrodermal activity, blood volume pulse (to calculate heart rate activity. i.e., beats per minute) and respiration rate. This work presents a comparison of the results provided by the analysis of physiological signals in reference to catecholamine, from an experimental task with 21 female volunteers receiving audiovisual stimuli through an immersive environment in virtual reality. Artificial intelligence algorithms for fear classification with physiological variables and plasma catecholamine concentration levels have been proposed and tested. The best results have been obtained with the features extracted from the physiological variables. Adding catecholamine’s maximum variation during the five minutes after the video clip visualization, as well as adding the five measurements (1-min interval) of these levels, are not providing better performance in the classifiers.

## 1. Introduction

Affective computing, the study, analysis, and interpretation of human emotional reactions by means of artificial intelligence [1], has become a hot topic in the scientific community. Possible applications include accurate neuromarketing techniques, more efficient human-machine interfaces and new wellness and/or healthcare practices, with innovative therapies for phobias and mental illnesses [2,3,4,5,6]. Recently, the prevention of violent attacks on vulnerable people by means of the early detection of fear or panic emotional reactions is under research in this area [7].

In affective computing, many research areas merge to provide efficient and accurate systems capable of classifying the emotion felt by a person. Apart from psychology, neuroscience and physiology, other disciplines are required to automate the emotion detection process as well as to allow in-depth data analysis and useful feedback.

Human emotions are the consequence of biochemical reactions in the brain. External stimuli are processed in certain brain regions such as the amygdala, insula and prefrontal cortex [8,9,10]. These areas activate the autonomic nervous system, which triggers physiological changes as an emotional response. From the global emotional response, we can distinguish conscious and unconscious processes. The cognitive component in the emotion obtains a high degree of consciousness and can feedback the physiological reactions chain.

The measuring and processing of these physiological reactions allow automatizing the emotion detection and classification process, known as affective computing. If this detection involves several sources of information, it is known as multimodal affective computing. Validity and corroboration issues have made physiological variables the most attractive to researchers. Multimodal recordings commonly used are Galvanic Skin Response (GSR), ElectroMyoGraphy (EMG) (frequency of muscle tension), Heart Rate (HR), Respiration Rate (RR), ElectroEncephaloGraphy (EEG), functional Magnetic Resonance Imaging (fMRI), and Positron Emission Tomography (PET) [11], even though behavioural measurements such as facial expressions, voice, movement, and subjective self-reporting can also be useful for experimental purposes. 

In this sense, some authors have related non-external physiological variables with emotional reactions [12]. For example, the levels of neurotransmitters in the brain or circulating catecholamines vary depending on a person’s emotional state, affecting activity of physiological variables. Although their measures are very invasive, the relation between physiological variable changes and the concentration of these molecules makes them interesting in some applications of affective computing. For example, in risk situations, this early detection of fear or panic emotions would trigger a protection response for the person in danger. To date, there is no study using catecholamine concentration in blood plasma for emotion detection that includes an experimental sample in humans, just theoretical studies.

The concentration of catecholamines is usually measured in urine to diagnose or rule out the presence of certain tumours such as pheochromocytoma or neuroblastoma because these tumours raise the levels significantly. However, in basal conditions, the levels are low and can be detected in blood by high-performance liquid chromatography (HLPC) techniques.

Continuous and autonomous measurement of these molecules is not available currently, but if they prove useful, wearable analysis devices could be designed and developed, similar to insulin micropumps [13].

In this work, a methodology and protocol are proposed to connect the elicitation of human emotions with the variation of plasma catecholamine concentration. For this first test, fear is chosen as the target emotion for two main reasons. On the one hand, the relationship between neurotransmitters and stress or fear is well documented in the literature, as they are responsible for the activation of the body’s fight or flight mechanisms. On the other hand, the protection of women against gender-based violence has been chosen as a target application. For this purpose, the objective is to be able to detect fear automatically so that an alarm is triggered to protect women in danger. Although there is already work in this area, so far only physiological variables have been used. In order to validate if the inclusion of catecholamine plasma concentration improves the results, an immersive virtual reality environment has been arranged to provoke realistic situations where the volunteer could have intense emotional reactions. Continuous monitoring of physiological variables, with a research toolkit system (for the sake of comparison with other affective computing research works), is connected with the virtual environment, as well as to an interface for the classification of the emotions elicited. The detection of emotions in humans through the plasma concentration of catecholamines has been analysed and compared with externally measured physiological variables, such as SKT, HR and EDA. The main obtained results are very positive with regard to physiological variables while they are not conclusive for the levels of catecholamine concentration in blood plasma.

The main contributions of this work can be summarized as:

- The design of a methodology for plasma catecholamine concentration measurement along with physiological variables under audiovisual stimuli for automatic fear detection.
- An experimental test involving 21 volunteers where dopamine, adrenaline and noradrenaline are measured along with blood volume pulse, skin temperature, galvanic skin response, respiration rate, and electromyography.
- An analysis of the data collected, including both physiological variables and catecholamine concentration separately and also combined.
- An implementation and comparison of three artificial intelligence methods for fear detection using the measurements collected in the experimental test in order to validate the convenience of including plasma catecholamine concentration in fear detection systems.

The rest of this paper is organized as follows: Section 2 provides a review of the state of the art regarding emotion theory, automatic emotion detection, and physiological response related to catecholamines and emotion. As result, we can formulate the hypothesis of this work. Section 3 describes the methodology used in this work for the experimental setup, including the sample description, the design of the study, the stimuli used, the labelling method, and the collected measurements. Section 4 presents the experimental results (for labelling, physiological variables and catecholamine concentration). Additionally, we present an artificial intelligence algorithm analysis in order to validate the hypothesis formulated previously. The discussion is presented in Section 5, and finally, Section 6 concludes the work. 

## 2. State of the Art: Emotions, Physiological Response and Affective Computing

### 2.1. Emotions

Emotions are fundamental for human beings since they play an important role in individual and social behaviour and mental processes, such as decision making, perception, memory, attention, etc. [14]. However, they have been partially ignored in the past, generally due to the difficulties they intrigue for experimental methodology.

The identification and classification of emotions for improving people’s lives have gained interest in recent years as several fields can take advantage of the results in this area [15,16,17]. such as mental health, human-machine interfaces, learning and teaching methods, video games or neuromarketing. In psychology, emotions are described as “psychological states that include three components: subjective personal experience, associated physiological response, and behaviours” [18,19].

**Figure 1:**
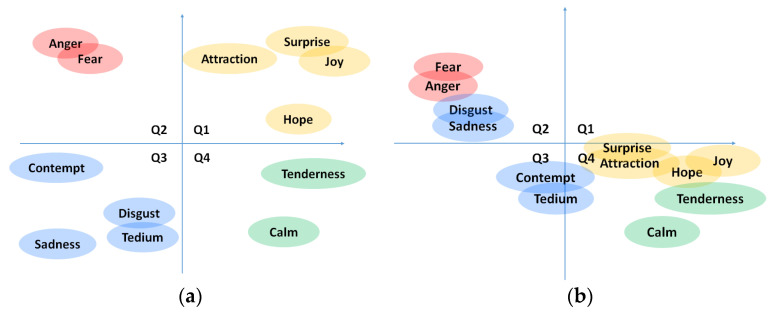
(**a**) Discrete emotion mapping in PA space in the literature. (**b**) Results extracted from Spanish study [28].

Within the literature and the state of the art in emotion identification and classification, there are two trends: (1) the classification of emotions as discrete elements, and (2) their inclusion in a continuous vector space. Within the first option, different classifications have been proposed. The first classification was presented by Ekman [20] using six basic emotions (happiness, sadness, disgust, fear, surprise, and anger). Since then, other classifications have been presented, adding emotions, or changing some of them [21,22]. Within the second option, we find the representation in the affective space. This consists of the multidimensional representation (usually within two or three axes) of the emotion so that the affective space becomes a continuous space in which every emotional state is represented by two or three coordinates. The most lately used space [23] proposes three dimensions (valence, arousal, and dominance). In this space, valence-pleasure (P) indicates positive or negative emotions; arousal (A) ranges from calm to high excitement levels; and finally, dominance (D) denotes the ability to control the emotion [24]. Several studies [25] of emotion classification use only a 2-dimensional space (PA space) using the valence and arousal axes previously described. That generates four quadrants in the space for locating emotions (Q1, Q2, Q3, and Q4). Some authors [26,27] have tried to place the discrete emotions in the quadrants according to the valence and arousal presumably experienced by each of them (see Figure 1a). Adding the third dimension (D) allows for differentiating discrete emotions sharing similar values in the PA space, such as fear and anger in Q2.

Both emotion classification systems present difficulties when applied to the automatic identification of emotions and their experimental validation. On the one hand, the use of discrete emotions is considerably biased by the sociocultural environment of the person [28], especially the background and the country of origin. In addition, there is reasonable dependence on the correct understanding of the description of the emotion or its nuances when identifying it [29]. In an attempt to address this, several emotions have been added to the list making it longer, but this also leads to problems for automatic emotion classification methods (as they add subtle differences in the responses). On the other hand, PAD affective space systems are often also related to the difficulty in understanding the three classification axes.

### 2.2. Emotion Detection

Affective computing has emerged to shed light on the gap where technology and emotions converge. One of the goals of this field is trying to model emotional response to a wide variety of stimuli by evaluating emotional states. These states become measurable regarding subjective self-reports, physiological variables and behaviour. 

The main elements involved in affective computing systems are the emotions theory [30] which connects human affective reactions to external stimuli, attending to intrinsic and extrinsic factors, with externally measurable physical and physiological changes; collecting data with smart sensors, first through emotion elicitation experiments in the lab and secondly through live in-the-wild monitoring; and the generation, training and integration of artificial intelligence algorithms in autonomous systems [3]. 

In affective computing, those changes are objectively measured in the person to determine the emotion felt. External (behavioural) aspects, such as facial expression, voice, movement, etc., are voluntary and biased through culture and society, making them difficult to apply to user-independent emotion detection. On the other hand, physiological changes (involuntary reactions) with an external effect (it is possible to measure them in a non-invasive way), have been preferred [31]. Typical variables used in affective computing include galvanic skin response, which increases linearly with a person’s level of arousal [32,33] electromyography (frequency of muscle tension), which is correlated with emotions of negative valence [34]; heart rate, which increases with negative valence emotions like fear [35,36]; respiration rate(how deep and fast the breath is), which becomes irregular with more aroused emotions like anger [37]; electroencephalography [38,39] and functional magnetic resonance imaging [40].

All these variables differ in many aspects, some of them are ease of measurement, which is related to how internal or external the target signal is; consciousness, because some variables can be consciously controlled and altered by the individual; and invasiveness, which means that some variables can be measured with low/high invasiveness for the individual. Many affective computing systems combine several variables in order to increase the performance of the application integrating solutions known as multimodal affective computing [41,42,43]. This allows combining several features from different sources making the automatic detection usually more complex but also with higher accuracy.

Intelligent algorithms should be trained with these measured physiological variables together with subjective perceived emotion during stimuli application. Among the different available options, we can feature according to the literature [44] those used in constrained devices as: Support Vector Machine (SVM) [45], K-Nearest Neighbours (KNN) [46] and Ensemble Methods (ENS) [47]. For training and research purposes, there are different databases compiling all these data for helping in the generation of affective computing systems [48,49].

The measurement of these physiological variables with wearable devices during daily life is associated with a high amount of noise due to interferences and users’ movements [50]. There are several works proposing solutions to eliminate or reduce this noise, through filters, algorithms, and even, fuzzy logic [51], but these techniques are expensive in terms of power consumption, the time required, and computation effort.

In order to try to overcome this problem, other variables could be tested in order to validate its inclusion pertinence. Among them, catecholamines’ presence in blood plasma, saliva or sweat could be an interesting option, even if its measurement is more invasive, as they could be more robust against artifacts. 

### 2.3. Chatecolamines in Emotion Detection

Since the first half of the 20th century, explanatory theories emerged to explain the physiological changes caused by stressful stimuli that altered the body’s homeostasis. These theories somehow evolved from the ”stress non-specificity” approach to the ”stress specificity” approach [52]. This means that the first theories of stress regarded this response relatively independent of the type of threat. Whether it was exposure to cold, haemorrhage or distressing emotional encounters, the stress response would be essentially the same [53]. However, recent data and observations indicate the probable existence of a variety of stressors with different targets and different effects on homeostasis [54]. These theories tend to explain the stress response by considering that it has a primitive type of specificity, with differential responses of the sympathetic nervous and adrenomedullary hormonal systems, depending on the type and intensity of the stressor perceived by the organism and interpreted in the light of experience [55]. The activation of the adrenomedullary hormonal system has been linked to glucoprivation and emotional distress such as fear. There is some evidence to confirm an accumulated association between noradrenaline and active escape, avoidance or attack, and a link between adrenaline and passive, immobile fear [56].

Catecholamines are hormones made in nerve tissue, the brain, and the adrenal glands. If they are found in the synapses of the nervous system, they are classified as neurotransmitters, and if they are found in the bloodstream, they are classified as hormones. The adrenal glands produce large amounts of catecholamines in response to acute stress or elevated arousal [57]. The main catecholamines are adrenaline (epinephrine), noradrenaline (norepinephrine) and dopamine. Catecholamines help the body to respond to stress or fear and prepare the body for “fight or flight” reactions [58]. This reaction to states of threat or high arousal results in a general discharge of catecholamines from three peripheral systems: the sympathetic branch of the autonomic nervous system, the adrenomedullary hormonal system and the autocrine/paracrine dopaminergic system. The activation of these systems favours the secretion of catecholamines into the bloodstream, where they trigger a cascade of physiological changes in peripheral tissues after binding to their receptors. Catecholamines increase heart rate, blood pressure, respiratory rate, muscle strength, and alertness. They also reduce the amount of blood going to the skin and intestines and increase blood going to major organs, such as the brain, heart, and kidneys [59]. 

**Figure 2:**
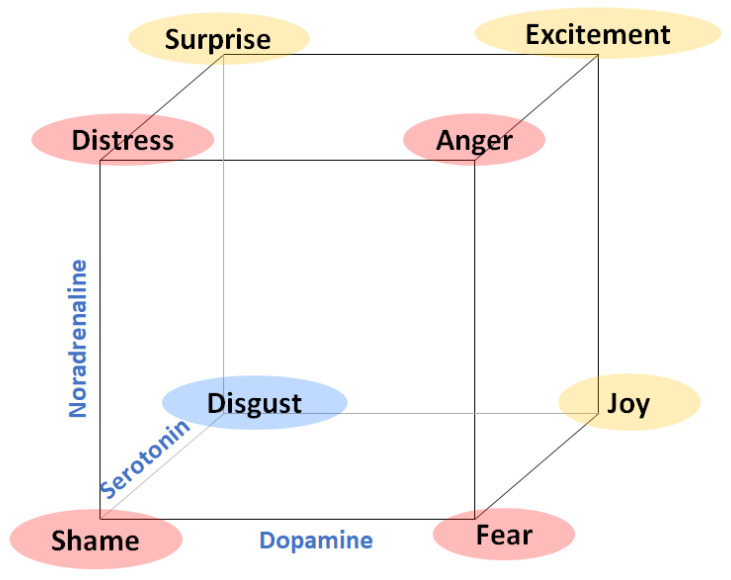
Loveheim cube showing correspondence among catecholamines and emotions (based on [12]).

Theoretical studies such as [12] propose that there is a direct relationship between neurotransmitter levels (dopamine, noradrenaline, and serotonin) and emotions. In this model, for example, fear is related to a combination of a low level of serotonin, a low level of noradrenaline and a high level of dopamine, (see Figure 2).

Loveheim’s study describes a theoretical framework that, if measurable, could improve multimodal affective computing systems for the automatic identification and classification of emotions. In fact, the study proposes to continue this research with a further experimental test that allows validating his proposal. Walker also proposes a theoretical framework that includes cortisol (a hormone produced in the adrenal gland) as an indicator related to fear and stress [60]. Again, this work suggested validating this framework with experimental tests. There are no results for catecholamines and human emotions experiments, although some previous tests have been performed in cats [61]. Directly measuring the presence of neurotransmitters is very invasive and nearly impossible on a day-to-day basis, so measuring catecholamines’ presence in blood plasma in an experimental setup in order to confirm whether there is a relationship between this presence associated with different emotional states is a good starting point for future developments in affective computing research. 

### 2.4. Hypotheses

Once the state of the art is reviewed, it can be stated that there is a lack of experimental studies that validate the relationship and convenience of using the concentration of plasma catecholamine in affective computing. So, in this work, the authors propose that:

- The emotional states of fear and no-fear can be discriminated through the plasma catecholamine concentration levels
- Using catecholamine concentration level improves the results for fear detection provided by the use of solely physiological variables.

If this hypothesis is proved correct, an automatic system for early detection of emotional states of fear can be implemented, reducing the effect of interferences and noise in the measured signals. Better protection for people in dangerous situations will be provided through the activation of early protective responses.

## 3. Material and Methods

In this section, we present the proposed methodology for data collection of both physiological variables and catecholamines in an immersive environment for emotion elicitation. Since the design of this experiment involves the extraction of blood samples for the analysis of catecholamines in blood plasma, and the number of samples cannot be high, fear has been chosen as the target emotion, since, as discussed in Section 2, it is highly related to the release of catecholamines. 

In addition, some considerations have to be taken into account. As stated before, one of the objectives of the authors is to apply multimodal affective computing to the protection of women victims of gender-based violence. For this reason, the sample of this study is entirely composed of women, and the proposed final application also influences the choice of one of the audio-visual stimuli, which is directly related to gender violence.

### 3.1. Sample of the Study

The study population consisted of 21 volunteers, all of them apparently healthy women. All of them were Spanish women, and healthcare workers. Study subjects were not allowed to perform strenuous exercise, smoke, eat some foods, or take drugs or some medicines (Table 1) at least 24 h before analysis, to avoid interference with catecholamines measurement. 

Main data of female volunteers are registered in Table 2. The mean age of the volunteers is 36. Only 5 of them had one child, and 13 volunteers were single. With regard to Body Mass Index (BMI), only 4 volunteers presented values between 25 and 30, overweight indicative. Finally, 4 volunteers are in their menopause. Some volunteers (6) were taking treatments for chronic illnesses (hypertension, chronic pain, heart failure, ulcerative colitis, anaemia, and diabetes).

The study conforms to the ethical principles outlined in the Declaration of Helsinki. Design of the study was approved by the Research Ethics Committee (REC) of Principe de Asturias Hospital with protocol number: CLO (LIB 10/2019). All participants received a detailed description of the purpose and design of the study and signed informed consent approved by the REC.

### 3.2. Design of the Study

The study consisted in measuring the physiological variables of a set of volunteers while they were watching a set of 4 emotion-related videos in an immersive virtual reality environment. Additionally, several blood extractions were performed after the visualization of three of these videos to analyse the plasma catecholamine levels (dopamine, adrenaline, and nor-adrenaline). Besides, after every video watching, the volunteer labelled the emotions elicited during the visualization.

Each participant fasted at least twelve hours before the experiment. Previously to the experiment, the participant filled in a form providing information such as personality traits, sex, age group, recent physical activity, or medication (which could alter the participant’s physiological response), self-identified emotional loads, and mood bias (fears, phobias, or traumatic experiences), summarized in Table 2. This information could be relevant and informative to the emotional reactions of the participants during the experiment, affecting their cognition, appraisal, and attention.

**Figure 3:**
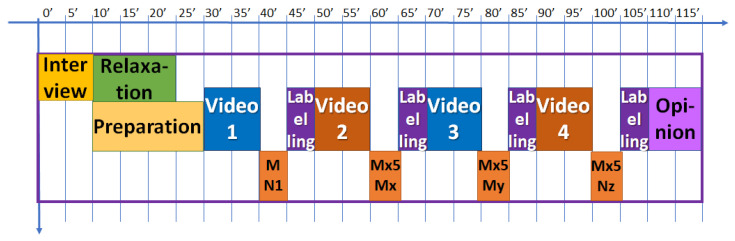
Schedule of the experiment for each volunteer.

**Figure 4:**
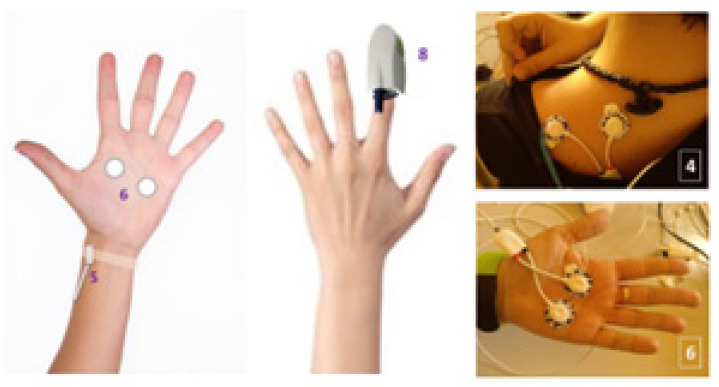
Electrodes and sensors position for experiment.

The experiment was designed to last globally 2 h. In Figure 3, the schedule of the experiment is shown. After the interview, filling in the questionnaire, and signing the informed consent, the test schedule and protocol were explained to every volunteer and some demo was performed in relation to the virtual reality environment. Then, the sensors for measuring the physiological variables were located. The BioSignalPlux^®^ research toolkit system was used to register the physiological variables evolution throughout the study, such as forearm skin temperature, galvanic skin response, finger blood volume pulse (BVP), trapezoidal electromyogram, and chest respiration. The system is placed in different locations in the volunteer’s body (arm, hand, chest, and finger), (Figure 4). These physiological signals were selected because they could be easily implemented in an inconspicuous and comfortable wearable device, avoiding any disadvantage to the user. There are smartwatches that already integrate BVP, GSR, and SKT sensors. Respiration and EMG could be integrated into a patch or band. This characteristic is mandatory for this type of application.

**Figure 5:**
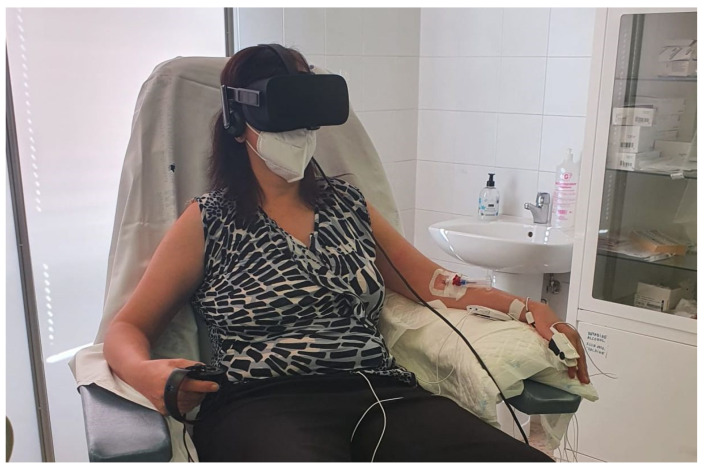
Volunteer ready to start the experiment.

Once explained how to handle the equipment to label each video, the nurse proceeded to put a via in the antecubital vein to extract blood samples at different time points of the study, at the beginning (basal point) and after each video (5 samples). Each subject watched four unexpected videos related to different emotions that had to be labelled according to what she was feeling at that moment. Just after finishing each video a blood sample was taken. After videos 2, 3 and 4, five samples were collected, separated 1 min each, to monitor the changes in catecholamine levels, (Figure 5).

### 3.3. Audiovisual Stimulus

Every subject watched four videos, two of them related to the emotion of fear, one related to calm and the other one related to joy. The schedule is Calm Fear Joy Fear. The order of fear-related videos is randomly set for each volunteer.

**Figure 6:**
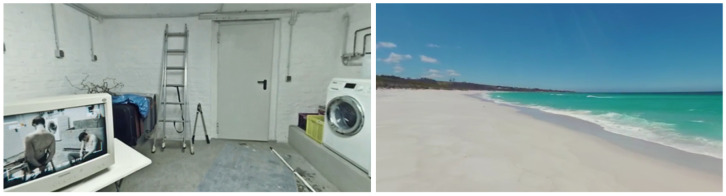
Screenshots for fear and calm video visualization.

The video clips used for the experiment were selected from the UC3M4Safety Database of audiovisual stimuli aimed to elicit different emotional reactions through an immersive virtual reality environment [62] (see Figure 6). Most of the clips were 360-degree scenes providing more realistic experiences. 

The Oculus™ Rift S Headset was used under an application built on Unity™ that connects the video clips projection to the physiological monitoring system and records the emotion labelling. The whole data recording system was initiated by the virtual reality environment that manages both video stimuli and sensor measurement. A TCP/IP port connection was created at the beginning of the trial to communicate with the OpenSignals application. The information storage was divided by scenes, meaning each file contained the information collected between two timestamps (start and end of each screen) set by the environment, thus enabling synchronization.

The four video clips were V1, V2, V3, and V4, aimed to provoke calm, fear (gender-based violence related), joy and fear, respectively.

- V1: “Nature”—calm
- V2: “Refugiado”—fear related to gender-based violence
- V3: “Don’t stop me now”—joy
- V4: “Inside chamber of horrors”—general fear

These videos obtained a very good unanimity in discrete emotion, higher in the case of women for the fear and joy clips while the mean and standard deviations in the PAD affective space dimension are also closer than expected for fear clips and for women, (Table 3). In this table, the discrete emotion labelled for every video is shown for the experiment detailed in [28], as well as the three dimensions of the PAD affective space. As it could be seen, V2 has a very high unanimity in the discrete emotion of fear in women, and also V4. Regarding PAD variables, the dispersion and the mean are complying with the expected ranges.

### 3.4. Labelling

**Figure 7:**
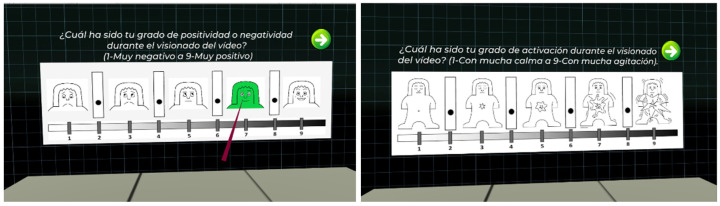
Labelling screen used in the experiment.

In order to try to overcome the problems related to labelling method mentioned above, in this work, we have decided to include both a discrete classification of emotions (joy, hope, surprise, attraction, tenderness, calm, tedium, contempt, sadness, fear, disgust, and anger), plus an indicator of emotional intensity to be able to detect more nuances, and the classification in the PAD affective space using the SAM methodology [63] (see Figure 7). As depicted in Figure 3, the labelling is carried out just after the blood sample collection.

### 3.5. Measurement of Dopamine, Adrenaline and Noradrenaline

We have carried out the determination of catecholamines in 3 mL of plasma by high-performance liquid chromatography (HPLC). Blood samples were collected in pre-chilled EDTA-treated tubes, in the morning after a 12-h overnight fast and resting period. As several samples had to be taken every few times after watching each video, a via was placed to assist sample collection from each point of the study. Plasmas were immediately separated, to prevent catecholamines degradation, by centrifugation at 2000× *g* for 15 min at 4 °C. After that, the plasma was collected in clean and pre-chilled tubes and then stored at −80 °C until measured. All plasmas were properly submitted to Reference Laboratory S.A. (L’Hospitalet de Llobregat, Barcelona, Spain) to measure by HPLC the adrenaline, noradrenaline and dopamine in each sample. 

Measurement of serotonin requires serum instead of plasma, needing the extraction of additional 5 mL blood samples from each volunteer. Apart from the extra cost, equivalent to measuring the other three catecholamines, the large number of samples required has prevented the authors from analysing the evolution of serotonin concentrations during the study.

## 4. Experimental Results

The experiments were performed from December 2020 to January 2021, on 12 and 9 volunteers, respectively.

### 4.1. Emotion Labeling

As it was already mentioned, emotional labelling is a complex task, not only because sometimes the target emotions are not the ones that are elicited to the volunteers, but also because of the terminology. 

For that reason, at first, it is important to analyse the distribution of the labels reported during the experiment and study how well the clips have been eliciting their target emotions.

**Figure 8:**
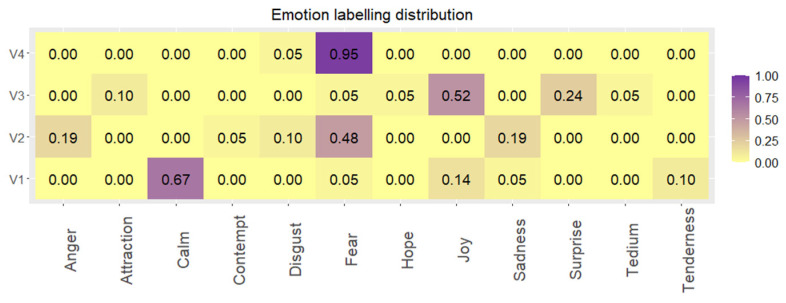
Emotion labelling distribution (0.00–1.00) between emotions reported by the volunteers w.r.t. each video clip visualized.

Taking into consideration discrete classification, (Figure 8), the clip targeting general fear emotion (V4) is the one with the highest agreement among the volunteers, 95% of them labelled it as fear. In the case of the clips of calm (V1) and joy (V3), a unique emotion does not obtain a clear majority; however, if the quadrants of PAD space are analysed, these videos show 76% and 90% of agreement, respectively.

On the other hand, V2 shows the highest dispersion, although fear is the most used label (48%), anger (19%), and sadness (19%) represent approximately 40% of the reported classifications. This scattering is mainly due to the scenes presented in the clip. As we have already found in previous works [28], gender-based violence videos elicit this variety of emotions depending on the volunteer’s perspective (first person or external).

As regards continuous labelling, independently from the dispersion found in discrete labelling, both fear clips are represented in their theoretical ideal position in the PAD space, low-valence, low-dominance and high-arousal corner. 

The same occurs with the calm and joy clips which are placed at spots of high-valence, medium-high dominance, and medium-low arousal, with the joy clip being slightly above in terms of arousal. 

**Figure 9:**
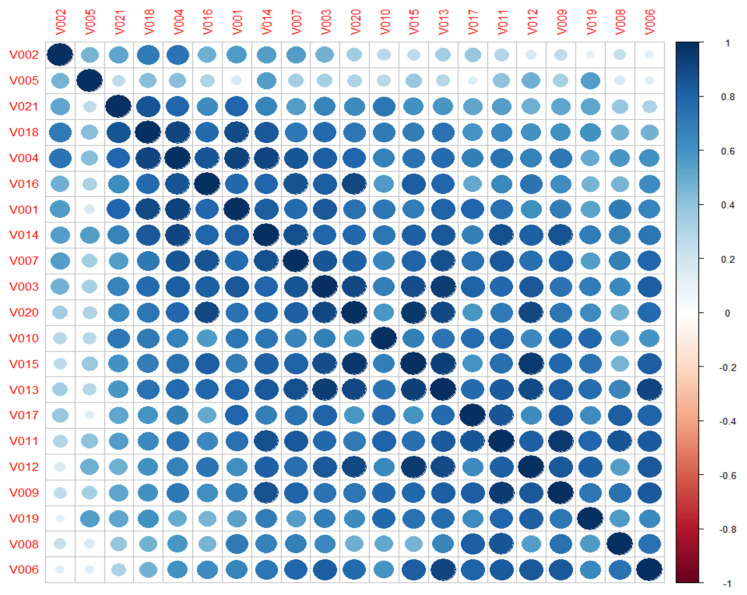
Correlation matrix between volunteers considering continuous reporting labelling.

Looking at previous results, and to observe the intercorrelation between volunteers when classifying all the clips, the correlation coefficient is computed considering all continuous reported labels. As result, a high positive relationship is obtained between all the volunteers, except for V002 and V005, who barely correlate with the rest, Figure 9. These results allow us to check that the emotions elicited are not only close to the original target (at least in the quadrant) but also inter-volunteer. 

### 4.2. Physiological Variables

From the physiological variables measured, the authors extracted features from the forearm skin temperature, skin conductance (GSR), finger blood volume pulse (BVP), and respiration. These variables have been measured throughout the whole experiment for every volunteer. First, a global analysis of the whole group of volunteers was carried out, for every video clip watched and, consequently, for every emotion. Later, temporal evolution of every physiological variable was also performed to find patterns of evolution during the visualization of the different emotion-related video clips.

#### 4.2.1. Median and Quartile Distribution of Extracted Features per Video Clip

This analysis has been performed on the measurements from all the volunteers, considering the target labels of emotion, normalizing every volunteer with respect to their own values.

Although Clip 2 (V2) and Clip 4 (V4) have the same fear label, V2 includes gender-based violence and the emotional reactions are very different from the reactions on V4, as it has been detailed in the previous section.

The extracted features from the physiological variables are Inter-Bit-Interval (IBI) and Heart Rate Variability (HRV) extracted from BVP, which are very related to the degree of arousal, and the phasic peaks of GSR and the mean of GSR, which have been identified with the variables that work better for artificial intelligent algorithms in affective computing. These features are computed in 60 s windows. 

**Figure 10:**
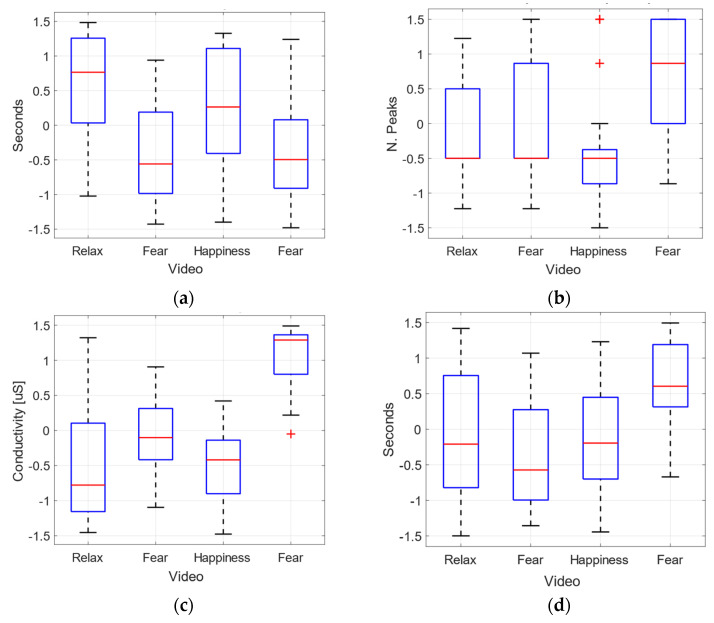
Normalized physiological features per video. (**a**) IBI. (**b**) number of phasic GSR peaks. (**c**) mean of GSR. (**d**) HRV rmssd.

As it can be observed in the Figure 10, the median and quartile distribution (box plots) IBI (a) and HRV (d) are the physiological features that better differentiate fear-related emotions, while the mean (c) and peaks (b) of GSR are clearly different for fear emotions (V4). Even, gender-based violence (V2) reactions are not distinguishable from calm or joy in terms of median values. 

The statistical analysis ANOVA on the features extracted from the physiological variables has provided some differences in the effect of different emotions elicited. In Table 4, the *p*-values for the comparison between videos are shown. We have observed significant values for the comparison between the effect of video clip V1 (calm) and video clips V2 and V4, for the mean of GSR. Additionally, there are significant differences in the effect of V1 and V4 for the IBI, and V3 and V4 for the number of peaks of GSR.

#### 4.2.2. Temporal Evolution of Physiological Variables

**Figure 11:**
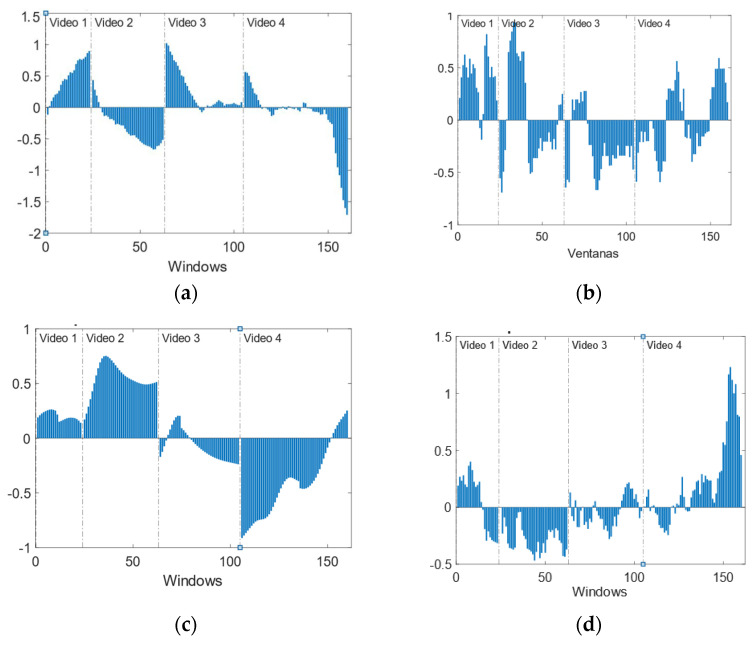
Temporal evolution of normalized features. (**a**) IBI. (**b**) Number of phasic GSR peaks. (**c**) Mean GSR. (**d**) HRV.

Temporal evolution analysis provides information about the evolution of the emotional state during the video. It should be noted that videos are labelled according to the prevailing emotion, but the same video could elicit more than one emotion, and the intensity could be non-homogeneous. This is a limitation of this type of experiment where continuous labelling is not possible. The result is dispersion/noise in the data, hindering their classification and modelling. Figure 11 shows the mean evolution of the four features used in the previous section.

**Figure 12:**
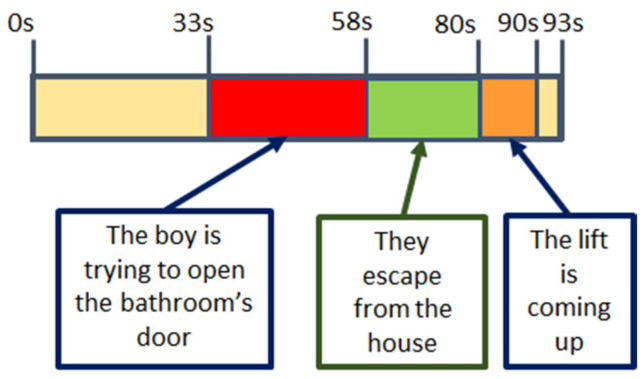
V02 main stressful events. “Refugiado” Diego Lerma 2014. Available at [62].

**Figure 13:**
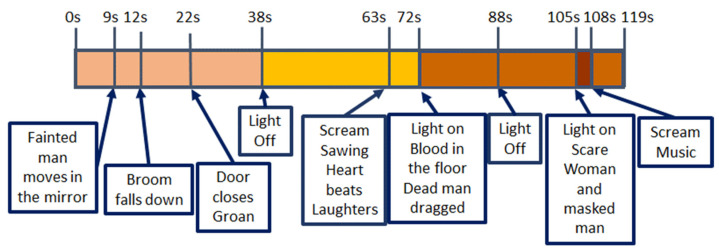
V04 main stressful events. “Chamber of horrors” Inside 360 VR Prod 2018. Available at [62].

The four videos present a high variation of the selected features, especially V4. These variations correlate with scenes in the videos. In Figure 12 and Figure 13, details on the scenes of both videos, V2 and V4, related to the fear emotion, are provided. As it could be seen, the most intense period of stress-fear in V2 is between seconds 32 and 58 when the boy is trying to open the bathroom’s door. In Figure 11, features extracted from physiological variables present a very different behaviour in this period of time that, in some cases, it is maintained untill the end of the video due to the empathizing effect with the escaping mother and boy. Until they discover the aggressor is not in the lift, second 90, the climax is maintained.

With regard to V4, all the scenes are stressful but peak instants are when lights go off (seconds 38 and 88) and there are screams or sudden hits/blows (seconds 12, 22, 63, and 105). The worst moment is when two people appear suddenly in front of the viewer, no-faced, with loud music and screams (105); all features show a change of behaviour around this final scare that has been under preparation right from second 63.

### 4.3. Catecholamine Concentration

**Figure 14:**
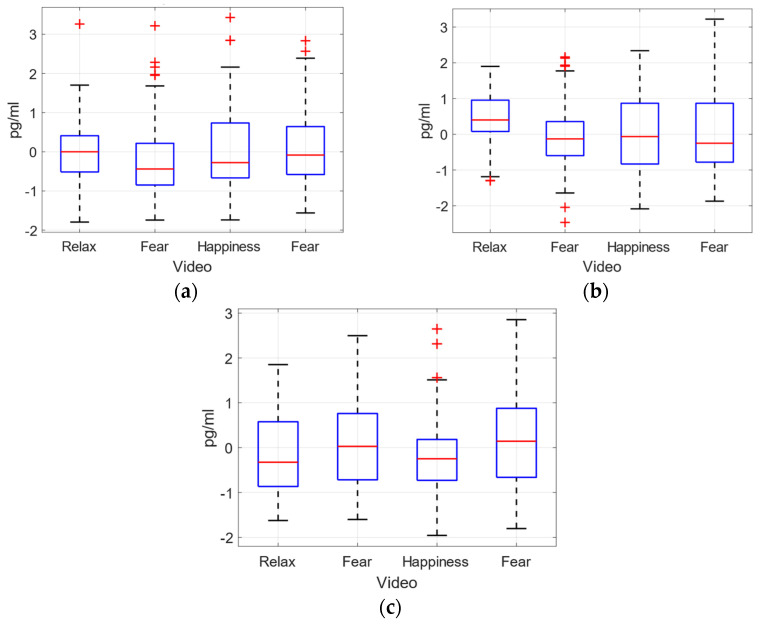
Normalized concentrations for dopamine, adrenaline and nor-adrenaline (**a**–**c**) for every video clip.

**Figure 15:**
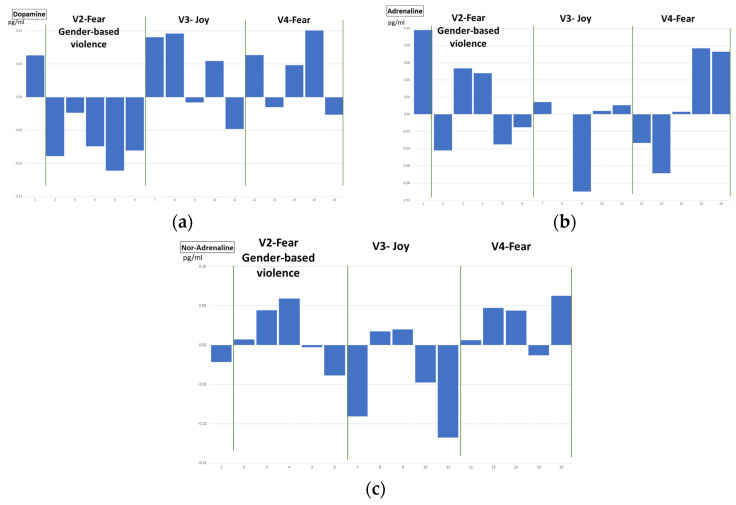
Temporal evolution of normalized concentrations for dopamine, adrenaline and nor-adrenaline (**a**–**c**) for every video clip, mean for all volunteers.

**Figure 16:**
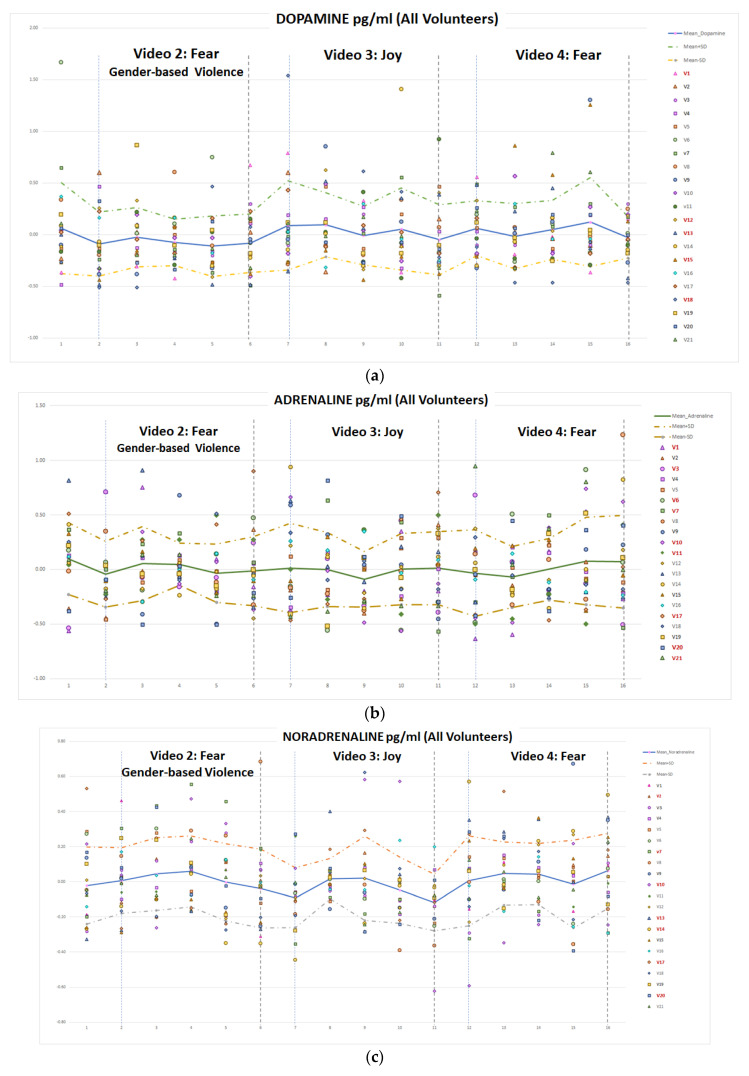
Temporal evolution of normalized catecholamine concentration for video clips 2, 3 and 4 for DA (**a**), A (**b**) and NA (**c**).

The concentration of adrenaline, dopamine and nor-adrenaline catecholamines, has been measured as detailed in Section 3, with the HPLC technique. In Table 5 the concentration values for these catecholamines are detailed per volunteer. A global analysis of these values has been performed to determine the relationship between the emotional reaction and these concentrations. First, the box plots of mean and quartile for every video clip were obtained, Figure 14. Second, to analyse the temporal evolution of these concentrations, temporal graphs were plotted, in Figure 15 and Figure 16.

#### 4.3.1. Catecholamine Concentration and Quartile Distribution

Data was collected per video clip, normalized per volunteer, and mean values were calculated for all the volunteers. 

The obtained values do not show differences in catecholamine concentrations for different emotion-related video clips, especially for adrenaline and dopamine. Furthermore, for these catecholamines (A and DA), the gender-based violence fear video clip (V2) presents very dispersed values, while the fear video clip (V4) provides higher dispersion just for dopamine, Figure 14.

The statistical analysis ANOVA of the plasma concentration level has not provided a clear difference between the effects of different emotions elicited for the three catecholamines measured. In Table 6 the *p*-values for the comparison between the videos are shown. No significant values have been obtained for any pair compared.

#### 4.3.2. Temporal Evolution of Catecholamines after Video Clip Watching

Figure 15 shows the temporal evolution of dopamine (a), adrenaline (b) and noradrenaline (c) for video clips V2, V3 and V4, related to fear (gender-based violence related), joy and fear, respectively. The graphs represent the concentration of catecholamines, per sample (five per video per volunteer), as well as the mean value (continuous line) and the mean plus/minus standard deviation (dashed lines) for all the volunteers. Catecholamine concentration values have been normalized with respect to the mean value of every volunteer. For the sake of clarity, and for comparison with respect to the behaviour of physiological variables, in Figure 15 the temporal evolution of the mean value (for all volunteers) has been plotted for the three catecholamines. Dopamine concentrations show a slightly different evolution after watching the video clips related to fear with gender-based violence than in those related to joy or fear, where a final drop can be appreciated, (Figure 15a). Adrenaline concentration shows a continuous rising tendency for the fear-related clip (V4) while for joy (V3), a stabilization is observed in the final samples (Figure 15b). In the gender-based violence clip (V2), the stressful/relieving situation may provoke a rise and a drop in the adrenaline’s concentration. Finally, in the noradrenaline’s concentration (Figure 15c), a similar evolution can be observed in V2 and V3 (fear with gender-based violence and joy) with a final drop in the normalized value, while V4 (intense fear) is not presenting the final drop, since the stressful situation continues to get even more stressful until the end of the clip.

### 4.4. Artificial Intelligent Algorithms

Considering our goal, which is to study the improvement that catecholamines measurements can bring to our fear/not-fear detection model and compare the results with physiological models, the data were normalized, reorganized, and grouped by clip for both data types to generate supervised techniques and evaluate performance metrics individually and together.

In this work the standardization selected is a modified version of self-dependent z-score; it consists of subtracting the mean value and dividing by the standard deviation of the complete experiment for each volunteer independently. 

The algorithms tested to classify the data were support vector machine (SVM), k-nearest neighbour (KNN), and ensemble (ENS). This selection was based on the target application, a wearable device with memory and computation power constraints. In addition, these methods are the most common ones used in the literature [44].

Each model’s hyper-parameters were tuned using Bayesian optimization to minimize the misclassification rate over iterations and supported by 5 k-fold cross-validation strategy. Specifically, the selected technique is a sequential model-based optimization, which has shown substantial improvements over combinational space approaches [64]. Besides, this training and validation scheme was based on previous works and results in [7]. The performance values presented were the mean validation results of 10 iterations. No testing was carried out due to the lack of data.

Table 7 shows the characteristics of the different models used to generate classifiers regarding the information source, number of features, and windowing. A detailed explanation is provided in the next subsections. Videos V02, V03, and V04 were considered in all cases.

The metrics selected to evaluate the classifiers’ performance are geometrical mean (Gmean) between Sensitivity (true positive rate, TPR) and Specificity (true negative rate, TNR) according to Equation (1). The TPR is the ratio between true positive (TP) and the sum of true positive and false negative (FN). The TNR is the ratio between true negative (TN) and the sum of true negative and false positive (FP).

(1) $$Gmean=\sqrt{(Sensitivity*Specificity}=\sqrt{\left(\frac{TP}{TP+FN}\right)*\left(\frac{TN}{TN+FP}\right)}$$

#### 4.4.1. Physiological Supervised Models

The classification of physiological data with supervised machine learning techniques is a common approach in affective computing due to the complex relationships that implies. The models presented in this work are user-independent because there is not enough data for user-dependent solutions. 

Two configurations were tested with the same number of features but with a different window size and overlapping. The features used are 22 for BVP, 7 for GSR, 6 for SKT, and 12 for respiration. The segmentation and windowing were applied following two strategies. Firstly, the configuration 1 used a 60 s window per video clip aiming to reduce data dispersion in the video. The second one has five windows per video, 20 s with 10 s overlap. This strategy helped algorithm training by providing more data and more temporal resolution; however, this could also lead to information redundancy.

The results in Table 8 showed that it is possible to classify the data between fear and no fear generally (Gmean above 0.5). The best performance was achieved by ENS (Adaboost) with the first model.

#### 4.4.2. Catecholamines Supervised Models

As in the physiological section, three algorithms KNN, SVM, and ENS (RandomForest) were applied (Table 9).

Firstly, each observation was associated with a clip and each feature to a sample of that clip, resulting in a data matrix of 63 rows (21 volunteers × 3 clips) and 15 columns (5 samples per clip × 3 catecholamines).

After achieving in almost all cases overfitted models or poor-quality metrics, a transformation of the data was applied to compute the maximum in-video variations, considering the sign positive if this variation was increasing (minimum previous maximum) or negative if it was decreasing (maximum previous minimum). This variable was obtained and then normalized for each catecholamine, resulting in a data matrix of 63 rows (21 volunteers × 3 clips) and 3 columns (1 maximum variation per clip × 3 catecholamines). 

As in previous models and mainly due to the lack of enough data and an imbalanced configuration, overfitted models were achieved and performance results worsened (Gmean values between 0.33 and 0.44) and showed the model would work randomly, such as flipping a coin. 

#### 4.4.3. Fusion Models

The data fusion applied followed two strategies based on physiological configurations. The first configuration was merged with the variation in plasma catecholamine concentration levels, per video clip, as explained previously (Model 5) and the physiological variables in a unique 60 s window. The second one used the plasma catecholamine concentration level directly, five samples per video clip. Each sample was paired with a 20 s physio window.

Table 10 shows the performance metrics obtained with the fusion models. The results were slightly worse than physiological models alone, i.e., the model was not learning from this data.

## 5. Discussion

The study conducted in this work presents four main results. First, a methodology and protocol have been defined to connect the elicitation of human emotions with the variation of plasma catecholamine concentration. An immersive virtual reality environment has been arranged to provoke realistic situations where the volunteer could have intense emotional reactions. A continuous monitoring of physiological variables, with a research toolkit system (for the sake of comparison with other affective computing research works), is connected with the virtual environment, as well as a labelling procedure for discrete emotions and continuous PAD affective space dimensions. These three elements have been presented in previous works by the authors [65]. The novelty added to this method is to determine whether a person’s emotions can be reliably recorded, assessing the differences or similarities between recording different physiological variables and measuring plasma catecholamine levels. The blood extraction must be performed after the video clip visualization to not interfere in the emotion elicitation but as soon as possible to detect the concentration peaks and valleys due to the emotion processed in the brain, which provokes a change in plasma catecholamine concentration. A pattern in the concentration variation has been looked for, as well as different classifiers, typical in affective computing, to determine the feasibility of using catecholamines for detecting fear emotions in a person.

Second, the emotion labels obtained during the study guaranteed the elicitation of the target emotions. The video clips selected were those with the best scores in terms of unanimity, in discrete and continuous emotions classifications, from the UC3M4Safety database [62]. The video clips’ durations were between 60 s and 119 s. The 21 volunteers labelled the emotion felt during the video clip visualization in a very close way to the target emotion, especially for video clips V04 (fear) and V01 (calm), while for the other clips, at least the PA quadrant is maintained, (Figure 8). Every video clip provoked the target emotions, and, except for two volunteers, every volunteer labelling process matched with the rest of them, (Figure 9). Therefore, the variation in the measures of physiological variables and plasma catecholamine concentration per video clip, whatever they were, can be associated with a specific emotion.

Third, the physiological variables measured during the study, and the features extracted from them (IBI, GSR number of peaks, GSR mean and HRV) present similar behaviour as in previous works [7,65]. Statistically representative differences between fear-related video clip V04 and joy and calm clips (V03 and V01) were found for the GSR mean, as well as between V01 (calm), V02 (fear related to Gender-based violence) and V04 (fear) for IBI. The classifiers applied to generate an artificial intelligence algorithm to detect fear emotional reactions present good results for windows of 20 s and 60 s, although the results were better for wider windows, and ENS model, with a True Negative Rate of 1 and a True Positive Rate of 0.83, (Table 8). 

It should be noted that the amount of data compiled during the experiment was large due to the sampling frequency (200 Hz), making easier the training and testing processes for affective computing tasks.

Finally, the plasma catecholamine concentration measurements provided data with apparently no connection with the emotion elicited. The ANOVA analysis provided no significant differences between the levels of catecholamines in blood plasma after visualizing the video clips of the different emotions. Besides, the clustering analysis (fear/no-fear emotions) on the data obtained from the 21 volunteers did not produce a valid result. Moreover, the classifiers selected as artificial intelligence algorithms to detect fear emotional reactions present poor-quality metrics, mainly due to the lack of enough data for training, testing and generalizing.

This problem of insufficient data on plasma catecholamine concentration (only five samples per video, i.e., per emotion) is difficult to solve. Even in an experimental study, the ethical research advises to not make volunteers suffer unnecessarily. Sixteen blood samples per session per volunteer, although taken through a via, while visualizing emotional intensive video clips within a virtual reality environment, are a fairly good number to test the hypothesis of the research work. In the literature, up to our knowledge, there is no similar study, with most of the proposals being theoretical hypotheses and/or based on analysing previous experimental results for other purposes.

However, the data obtained should have provided some patterns of responses to different target emotions and, although in the temporal evolution of the concentration levels of adrenaline and nor-adrenaline a similar behaviour can be observed after both V02 and V04 fear-related clips, neither statistically significant relations have been found nor affective computing classifiers provided good results.

It is true, that the plasma catecholamine levels are altered by the effect of some foods, drinks, and medicines or drugs, as well as by strong physical exercise and/or recent intense stressful episodes. Amines found in banana, avocado, walnuts, beans, cheese, beer and red wine can modify the concentration of these hormones in the blood. Additionally, foods/drinks with cocoa, coffee, tea, chocolate, liquorice, or vanilla, as well as drugs (nicotine, cocaine and ethanol) and medicines (aspirin, tricycle antidepressants, tetracycline, theophylline, blood pressure control agents, and nitro-glycerine) have similar effects. 

Besides, the emotional response is altered by prior experiences during a lifetime, and so does the emotional response to stress and the conditioned response to fear. Traumatic stress-induced fear memories may affect the physiological response and plasma catecholamine levels. There is strong evidence supporting that central catecholamines are involved in the regulation of fear memory, by activation of the sympathetic nervous system with elevated basal catecholamine levels are common in patients suffering from post-traumatic stress disorder (PTSD). 

In the study presented, attention is paid to the activity of the volunteers before the experiment, as well as the different substances taken and, also, previous traumatic stressful experiences.

Although we previously informed about the recommendations, the volunteers reported the following data. With regard to medicines as regular treatment, six volunteers reported five chronic diseases: diabetes mellitus (1), hypertension (2), cardiac failure (1), ulcerative colitis (1), anaemia (1), and chronic pain (1). Additionally, one volunteer was taking contraceptives. On the other hand, four volunteers were taking ibuprofen or another type of anti-inflammatory drugs for the two days prior to the experiment. Respect to avoiding stimulants in food, drinks and drugs in the 24 h prior to the experiment, 13 volunteers took coffee or tea in that period of time, and one volunteer drank alcohol. Additionally, three of them ate citric fruits in that period.

Only four volunteers (v06, v11, v13, v19) exactly complied with the recommendations with regard to avoiding stimulant foods, drinks and drugs; and did not take any medication. They were young women with ages 23, 30, 29, and 23, respectively. Likewise, three volunteers (v01, v04, and v17) only had a coffee, complying with the rest of the recommendations, and did not take any medication either. Their ages were 21, 55, and 24 respectively. There are seven volunteers that only took a coffee and medicaments not presenting differences in the levels of catecholamine concentrations (v02, v05, v09, v12, v14, v15, and v20). In summary, we can consider that 14 volunteers were fully compliant and 7 could have some objection with respect to regular catecholamine activity.

Regarding prior stressful experiences, or specific fears, seven volunteers reported some previous traumas that activate themselves in situations like video clips V02 and V04, (v01, v03, v04, v12, v15, v16, and v20). Two of them identified as gender-based violence victims. However, the evolution of their plasma catecholamine concentration levels were not different from the other volunteers’, (Figure 15 and Figure 16).

Apart from the extrinsic and intrinsic factors that can be affecting the results of the study, the authors wish to highlight the low levels of the concentration of these catecholamines present in the blood plasma. We tested the technique ELISA that produced worse results in terms of sensitivity of these catecholamines. Nine women volunteers followed a similar experimental study, and 15 blood samples per volunteer were analysed with ELISA kits. 

With respect to the hypothesis stated in this work, the measurement of the levels of dopamine, noradrenaline and adrenaline concentration in blood plasma is neither providing better classifications nor a more accurate differentiation of fear-emotion reactions in women.

## 6. Conclusions

In this work, a methodology and a protocol have been proposed to connect the elicitation of human emotions with the variation of plasma catecholamine concentration. For them, an immersive virtual reality environment has been arranged to provoke realistic situations where the volunteer could have intense emotional reactions. A continuous monitoring of physiological variables, with a research toolkit system (for the sake of comparison with other affective computing research works) was connected to the virtual environment, as well as a labelling procedure for discrete emotions and continuous PAD affective space dimensions. 

Using this methodology, an experimental study with 21 volunteers has been conducted, using fear as a target emotion, thus provoking fear and non-fear while measuring physiological variables and extracting blood samples after the visualization of every video stimulus. In this first study, 16 blood samples have been extracted per volunteer; 1 for basal measure and 5 after the three emotion-related video clips (fear (gender-based violence related), joy and fear). These samples have been extracted in 1-min intervals after the visualization of the video clip. Along with the blood sample for catecholamine plasma analysis, physiological variables have been measured during the visualization of the video clips. Skin temperature, galvanic skin response, blood volume pulse, respiration, and Trapezoidal Electromyogram were the selected variables, measured with a commercial research toolkit.

Additionally, the emotion labelling for every video clip by all the volunteers has been analysed and there is a high degree of agreement in the discrete emotion, which was even better in the PAD affective space dimensions, especially for fear-related video V04. Therefore, we can affirm that the selected video clips are meaningful for the experiment.

The results for the evolution of the features extracted from the physiological variables, as well as an ANOVA statistical analysis, are in accordance with previous works. Differences between features measured during fear-related and during calm and joy-related video clips have been found for the mean of GSR (60 s windows). Additionally, differences have been found between calm-related and fear/gender-based-violence fear-related video clips for the IBI (for heart rate,). Furthermore, the temporal evolution of these features has been analysed and correlated with the fear-related video clips, identifying precise moments where the features’ behaviour can be associated with the scene development. 

We can conclude that there are no significant *p*-values (ANOVA statistical analysis performed) that allow differentiating the emotion elicited using only the evolution of the plasma catecholamine concentration levels as a variable. Additionally, the temporal evolution of these levels has been analysed, not identifying precise patterns for fear-related video clips different from the joy-related video clip.

Finally, artificial intelligence algorithms for fear classification with physiological variables and plasma catecholamine concentration levels (separately and together) have been tested. The best results have been obtained with the features extracted from the physiological variables. Adding the maximum variation of catecholamines during the five minutes after the video clip visualization, as well as adding the five measurements (1-min interval) of these levels, do not provide better performance in the classifiers.

The small number of samples together with the low concentration of catecholamines in blood plasma make it not possible to use these data for machine learning techniques for fear classification in this experiment.

Finally, we can state that research on this topic should continue considering the following future actions:

1. Although it is true that the results of this study show that the measurement of catecholamine concentration does not improve the detection and identification of emotions, it would be desirable to have a larger sample of volunteers in order to detect patterns of variation in this concentration that validate this conclusion.
2. Following Lovehëim’s theory work, adding the measurement of blood serotonin concentration would be recommendable since it could allow us to improve the classification of fear from joy, which are both emotions with a high theoretical degree of activation. For this study, although its inclusion was considered, adding the serotonin measurement entailed the use of another analysis technique, which meant extracting twice as many samples from each volunteer, which was not recommended from an ethical point of view.
3. In the search for non-invasive emotion detection systems, it would be interesting to analyse the effect of the concentration of catecholamine in sweat (cortisol) or in saliva (alpha-amylase). If significant differences were found, it would be possible to include these variables in automatic emotion detection systems design.
4. However, in the search for any other extra information, instead of clustering fear and not-fear emotions, a behaviour pattern for each volunteer was examined according to Khrone [66] which suggests that there are two main strategies in stress reaction: vigilance and avoidance. From an unsupervised standpoint and after applying k-means algorithms four clear groups were observed, two of them being a symmetrical representation of the other two. In two of the groups, the third clip contains a negative variation, which is below the other two clips. On the other hand, the other two groups have a peak in the third clip (V3) which is above the values representing the other two videos.

## Figures and Tables

**Table 1:** Foods, drinks, and drugs can interfere with the analysis of catecholamines.

Food	Drinks	Drugs	Medicines
Cocoa	Coffee	Amphetamine	Paracetamol
Citric Fruits	Tea	Caffeine	Phenoxybenzamine, phenothiazine
Walnuts	Chocolate	Nicotinic Acid	Levodopa
Beans	Beer	Cocaine	Monoamine oxidase inhibitors
Avocado, Banana	Red wine		Reserpine
Vanilla			Pseudoephedrine

**Table 2:** Characteristics of women volunteers.

Parameter	Mean ± Std Deviation (SD)/Nb.
Age (year)	36.19 ± 13.43
Weight (kg)	61.20 ± 8.68
Height (cm)	164.29 ± 5.09
BMI (kg/m^2^)	22.75 ± 3.56
Food, drinks, drugs	Citric fruits (3), coffee (11), tea (2) and alcohol (1)
Medicines reported	Analgesic (5), chronic illness treatment (3), contraceptives (1), and vitamin (1)
Stress situation	5
Intense exercise	2

**Table 3:** Emotional Labelling of the video clips used in the experiment [28].

Video Clip	Target Emotion	Duration	Unanimity (Discrete) Men Women		PAD (Mean/SD) Men Women	
V1	Calm	60 s	78%	74,4%	V: 7.3 (1.7) A: 2.1 (1.1) D: 6.8 (1.8)	V: 7.7 (1.7) A: 2.0 (1.7) D: 6.6 (2.4)
V2	Fear Gender-based violence	93 s	62.1%	93.2%	V: 2.5 (1.8) A: 7.1 (1.2) D: 4.2 (1.7)	V: 1.7 (0.7) A: 7.7 (0.9) D: 3.4 (1.6)
V3	Joy	101 s	71.9%	83.3%	V: 7.3 (1.6) A: 4.6 (2.1) D: 6.6 (2.0)	V: 7.8 (1.3) A: 4.5 (2.2) D: 7.2 (1.9)
V4	Fear	119 s	75.0%	84.2%	V: 2.9 (1.7) A: 6.6 (1.7) D: 4.3 (2.3)	V: 2.7 (1.6) A: 6.9 (1.7) D: 4.3 (2.2)

**Table 4:** *p*-values results from Kruskal-Wallis one-way ANOVA test for physiological data grouped by video clip.

Group A	Group B	GSR_mean	GSR_npeaks	HRV	IBI
V1	V2	0.22291	0.99970	0.63272	** 0.00155
V1	V3	1	0.75762	0.97703	0.66276
V1	V4	** 1.82 × 10^−7^	0.03096	0.07931	** 0.00119
V2	V3	0.22578	0.70152	0.86245	0.06034
V2	V4	* 0.00163	0.04035	** 0.00196	0.99989
V3	V4	** 1.89 × 10^−7^	** 0.00111	0.02655	0.05052

NOTE: Significant codes: ‘**’ 0.001 ‘*’ 0.01 ‘ ’ 0.05.

**Table 5:** Plasma catecholamine concentration levels for every volunteer for every sample (pg/mL), for adrenaline (A), dopamine (DA) and noradrenaline (NA).

		**Volunteer 1**			**Volunteer 2**			**Volunteer 3**			**Volunteer 4**			**Volunteer 5**			**Volunteer 6**			**Volunteer 7**		
	**Sample**	**A**	**DA**	**NA**	**A**	**DA**	**NA**	**A**	**DA**	**NA**	**A**	**DA**	**NA**	**A**	**DA**	**NA**	**A**	**DA**	**NA**	**A**	**DA**	**NA**
Video 1 - Basal	1	12	11	274	15	12	503	16	9	292	45	13	309	31	13	338	32	29	566	41	52	331
Video 2: Refugee (Fear GBV)	2	47	11	492	13	25	480	59	9	434	42	37	346	16	13	270	29	10	454	30	24	538
	3	48	12	379	23	16	614	32	11	455	44	22	371	26	11	336	22	11	579	37	34	591
	4	29	10	287	22	17	456	29	10	500	45	21	411	32	15	249	26	12	467	40	27	642
	5	30	11	360	23	11	604	32	8	520	32	21	310	29	11	294	31	19	500	25	20	601
	6	23	29	232	32	16	547	43	13	434	42	28	424	29	17	231	40	10	435	32	16	491
Video 3: Queen (Joy)	7	17	31	335	19	25	445	21	9	373	26	30	362	33	11	247	23	10	415	25	31	267
	8	21	20	302	24	10	569	38	9	396	46	29	368	24	22	234	12	11	451	49	28	376
	9	22	23	344	14	11	633	23	12	375	40	32	415	30	13	238	37	8	402	21	32	337
	10	37	11	300	28	14	542	50	9	363	30	17	313	38	18	237	12	10	446	43	49	371
	11	22	13	302	33	18	469	21	9	351	40	26	410	38	22	201	19	9	333	13	13	376
Video 4: Inside de chamber of horror (Fear)	12	10	27	284	27	11	492	58	9	289	46	26	413	28	16	300	14	13	474	21	47	279
	13	11	14	374	20	16	520	37	9	402	48	17	442	30	14	298	41	8	451	28	40	414
	14	32	17	410	29	15	558	28	11	330	46	21	415	36	13	273	21	12	446	45	34	343
	15	42	11	280	25	14	595	30	9	426	39	30	397	27	14	264	52	9	338	27	41	267
	16	20	20	368	19	15	623	17	13	450	31	21	361	26	15	271	29	11	478	14	37	293
		**Volunteer 8**			**Volunteer 9**			**Volunteer 10**			**Volunteer 11**			**Volunteer 12**			**Volunteer 13**			**Volunteer 14**		
	**Sample**	**A**	**DA**	**NA**	**A**	**DA**	**NA**	**A**	**DA**	**NA**	**A**	**DA**	**NA**	**A**	**DA**	**NA**	**A**	**DA**	**NA**	**A**	**DA**	**NA**
Video 1 - Basal	1	19	15	363	27	16	144	28	14	475	23	13	233	27	12	233	39	31	225	24	14	315
Video 2: Refugee (Fear GBV)	2	26	9	437	17	11	129	20	9	406	17	13	238	23	17	229	16	16	242	14	12	370
	3	18	9	475	13	11	114	34	16	289	28	19	239	21	18	212	41	34	268	12	14	387
	4	20	18	492	37	16	137	22	13	576	28	11	212	27	9	253	20	24	278	13	15	449
	5	17	10	481	11	12	108	27	13	521	33	16	270	25	8	183	17	16	256	14	9	280
	6	13	9	642	21	17	95	16	8	419	28	18	299	14	9	239	14	16	244	16	10	279
Video 3: Queen (Joy)	7	16	8	311	35	17	125	42	11	421	22	16	319	31	10	210	35	20	241	33	11	239
	8	15	10	375	29	33	107	25	20	370	16	16	267	29	22	239	22	47	468	19	13	458
	9	12	9	375	23	13	119	13	14	619	30	22	277	20	9	235	19	33	240	11	9	328
	10	19	15	233	23	20	108	11	10	615	13	9	250	26	12	197	26	42	348	14	31	420
	11	20	12	243	12	13	100	22	11	148	33	30	256	35	19	226	25	43	303	19	9	416
Video 4: Inside de chamber of horror (Fear)	12	22	9	380	21	12	114	14	11	160	11	15	228	35	18	178	15	46	452	18	9	675
	13	13	12	370	17	18	121	13	21	255	12	12	247	20	9	253	23	38	429	13	12	423
	14	21	13	338	18	20	141	35	11	296	17	12	229	23	14	280	16	45	453	11	15	530
	15	14	11	246	26	41	212	44	17	476	11	11	217	23	13	293	17	27	333	17	13	554
	16	43	14	322	27	13	171	41	16	295	31	14	251	30	16	238	16	18	457	31	11	643
		**Volunteer 15**			**Volunteer 16**			**Volunteer 17**			**Volunteer 18**			**Volunteer 19**			**Volunteer 20**			**Volunteer 21**		
	**Sample**	**A**	**DA**	**NA**	**A**	**DA**	**NA**	**A**	**DA**	**NA**	**A**	**DA**	**NA**	**A**	**DA**	**NA**	**A**	**DA**	**NA**	**A**	**DA**	**NA**
Video 1 - Basal	1	49	13	153	38	20	447	31	15	288	29	15	333	33	16	627	15	16	359	23	18	312
Video 2: Refugee (Fear GBV)	2	33	10	147	31	17	609	15	18	138	24	10	297	28	12	710	22	20	332	17	11	324
	3	43	15	187	24	14	539	26	14	150	26	10	286	26	25	704	12	11	438	24	13	302
	4	38	19	186	34	17	481	20	12	160	21	16	388	26	12	630	25	10	285	24	13	407
	5	29	13	171	39	12	586	29	17	143	35	30	259	23	14	462	12	17	300	16	14	335
	6	33	11	159	31	12	519	39	18	143	17	22	284	27	11	552	19	14	278	16	11	318
Video 3: Queen (Joy)	7	33	13	186	43	15	516	11	21	167	31	52	288	16	15	411	18	16	391	12	18	325
	8	48	15	204	40	10	498	14	13	223	21	19	375	13	15	583	44	14	331	13	16	347
	9	37	10	228	46	19	496	15	16	243	25	33	578	30	11	606	27	11	220	15	19	247
	10	27	14	211	33	14	643	30	15	147	19	29	383	25	11	575	36	14	233	14	16	270
	11	39	11	191	37	11	624	35	12	166	19	16	323	36	12	516	17	13	310	14	11	301
Video 4: Inside de chamber of horror (Fear)	12	44	14	255	31	17	508	12	17	239	30	18	306	27	15	604	14	19	395	41	20	367
	13	45	33	199	39	19	433	17	10	285	22	11	444	22	13	483	35	16	387	22	11	345
	14	47	28	282	30	14	594	11	11	192	19	11	418	36	10	603	15	18	240	29	29	298
	15	23	40	234	27	14	387	13	12	203	20	19	280	41	14	600	33	18	187	38	26	312
	16	35	20	259	26	14	368	21	14	222	19	11	435	30	11	496	34	15	282	21	15	401

**Table 6:** *p*-values results from Kruskal-Wallis one-way ANOVA test for catecholamine concentration data grouped by video clip.

Group A	Group B	Adrenaline	Noradrenaline	Dopamine
V1	V2	0.82591	0.90859	0.62776
V1	V3	0.65790	0.99983	0.97443
V1	V4	0.76604	0.95005	0.99913
V2	V3	0.95784	0.56652	0.53611
V2	V4	0.99743	0.99573	0.25316
V3	V4	0.98951	0.71117	0.95883

**Table 7:** Characteristics of each configuration.

Nb. Configs	Physio	Cat.	Observations	Features	Window Size	Overlap
1	✓	-	63	47	60 s	-
2	✓	-	315	47	20 s	10 s
3	-	✓	63	15	-	-
4	-	✓	63	3	-	-
5	✓	✓	63	48	60 s	-
6	✓	✓	315	48	20 s	10 s

**Table 8:** Performance metrics for physiological configurations.

Nb. Config	Algorithm	G. Mean	TPR	TNR
1	SVM	0.59	0.83	0.51
	KNN	0.74	0.83	0.67
	ENS	0,91	0.83	1.00
2	SVM	0.56	0.86	0.45
	KNN	0.64	0.83	0.50
	ENS	0.74	0.83	0.66

**Table 9:** Performance metrics for catecholamines models.

Nb. Config	Algorithm	G. Mean	TPR	TNR
3	SVM	0.49	0.47	0.55
	KNN	0.53	0.51	0.58
	ENS	0.45	0.47	0.50
4	SVM	0.33	0.29	0.73
	KNN	0.37	0.25	0.64
	ENS	0.44	0.42	0.53

**Table 10:** Performance metrics for merged models.

Nb. Config	Model	G. Mean	TPR	TNR
5	SVM	0.57	0.88	0.46
	KNN	0.72	0.81	0.65
	ENS	0.90	0.81	1.00
6	SVM	0.52	0.88	0.41
	KNN	0.64	0.82	0.52
	ENS	0.74	0.82	0.67

## Data Availability

Not applicable.
